# SUBJECTIVE COGNITIVE CONCERNS AND 5-YEAR NEUROPSYCHOLOGICAL TRAJECTORIES IN PARKINSON’S DISEASE

**DOI:** 10.1093/geroni/igad104.3131

**Published:** 2023-12-21

**Authors:** Britney Luu, Mary Ellen Garcia, Katherine Bangen, Douglas Galasko, Kelsey Thomas

**Affiliations:** San Diego State University, San Diego, California, United States; California State University, San Bernardino, San Bernardino, California, United States; University of California, San Diego School of Medicine, San Diego, California, United States; University of California, San Diego School of Medicine, San Diego, California, United States; University of California, San Diego School of Medicine, San Diego, California, United States

## Abstract

Subjective cognitive concerns (SCC) are often associated with Alzheimer’s disease (AD)-related cognitive declines in cognitively unimpaired (CU) individuals. However, it is unknown whether the same is true for CU individuals with Parkinson’s disease (PD). Therefore, this study examined relationships between SCC and 5-year neuropsychological trajectories in CU individuals with newly-diagnosed PD (mean age=60.45 years; 35.2% female) in the Parkinson’s Progression Markers Initiative (PPMI) cohort. Participants were classified as having SCC (n=52) or no SCC (n=204) based on participant or caregiver report for item 1 on the Unified Parkinson’s Disease Rating Scale, part 1. First, baseline cerebrospinal fluid (CSF) biomarkers were examined by SCC group. Next, linear mixed effects models, adjusting for age, education, sex/gender, APOE genotype, psychiatric factors, and motor symptoms examined 5-year trajectories of individual neuropsychological measures by SCC status. Relative to participants without SCC, those with SCC had worse levels of CSF tau (p=.011), p-tau (p=.016), and p-tau/amyloid ratio (p=.003), but not amyloid or alpha synuclein. SCC was associated with faster rates of decline in learning (β=-.059, p=.018), memory (β=-.050, p=.035), and processing speed (β=-.052, p=.031), but not in semantic fluency, visuoperceptual abilities, working memory, or on a global cognition screening measure. Overall, SCC was associated with learning/memory and processing speed declines, but not other measures that may be more PD-specific (e.g., working memory, visuoperceptual). Given the elevated AD biomarkers, SCC may be particularly related to early changes due to co-occurring AD and PD pathology. Future work should investigate longitudinal AD and PD-related biomarker trajectories by SCC status.

